# Global Spatiotemporal Dynamics of African Swine Fever: An Integrated Multi-Scale Spatial and Time-Series Analysis

**DOI:** 10.3390/v18060618

**Published:** 2026-05-28

**Authors:** Renfeng Li, Jiaxin Jiang, Yunshi Liu, Wenyan Cao, Peng Li, Hongxuan He

**Affiliations:** 1 College of Animal Science and Veterinary Medicine, Henan Institute of Science and Technology, Xinxiang 453003, China; lirenfeng@sina.com (R.L.); jiangjiaxin@stu.hist.edu.cn (J.J.); caowenyan@stu.hist.edu.cn (W.C.); 2 Henan Provincial Key Laboratory of Zoonoses of Companion and Wild Animals, Henan Institute of Science and Technology, Xinxiang 453003, China; 3 School of Biological Engineering, Xinxiang University, Xinxiang 453003, China; 4 National Research Center for Wildlife-Borne Diseases, Institute of Zoology, Chinese Academy of Sciences, Beijing 100101, China

**Keywords:** African swine fever, spatiotemporal epidemiology, time series analysis, transboundary transmission

## Abstract

African swine fever (ASF) poses a persistent and escalating threat to global swine production. To comprehensively characterize its global spatiotemporal dynamics from 1996 to 2025, we developed an integrated framework combining multi-distance spatial analysis and advanced time series forecasting, utilizing a dataset of 57,253 outbreak records. Our findings reveal a clear divergence in transmission patterns: wild boar accounted for approximately 70% of outbreaks and predominantly sustained transmission in Eastern Europe, whereas domestic pig outbreaks were largely concentrated in Southeast Asia. A pronounced epidemiological shift occurred between 2017 and 2020, during which ASF spread transitioned from a predominantly north–south axis linking Africa and the Caucasus to a broad east–west expansion across Eurasia, coinciding with rapid dissemination throughout Asia. In the Northern Hemisphere, ASF outbreaks exhibited a bimodal seasonal pattern, with peaks observed in January–March and July–August. Comparative forecasting analyses demonstrated that machine learning approaches consistently outperformed both traditional statistical and deep learning models. Among these, the random forest algorithm achieved the highest predictive accuracy, surpassing SARIMA, Prophet, XGBoost, and GRU. Collectively, these findings underscore the pivotal role of wild boar in maintaining global ASF transmission and highlight the necessity of integrated surveillance at wildlife–livestock interfaces. Furthermore, they support the application of machine learning-based approaches for improving early warning systems and enhancing the effectiveness of global ASF control strategies.

## 1. Introduction

ASF is a severe, notifiable hemorrhagic disease of swine caused by the African swine fever virus (ASFV), characterized by exceptionally high mortality in affected populations [1]. Clinical manifestations typically include high fever, cutaneous and internal hemorrhages, lethargy, anorexia, and sudden death [2]. As a paramount threat to the global swine industry, ASF’s severe socio-economic impact has led the World Organisation for Animal Health (WOAH) to classify it as an animal disease subject to statutory reporting, while China categorizes it as a Class I animal infectious disease [3,4]. The global footprint of ASF has expanded dramatically to encompass over 70 countries and regions, reflecting an accelerating transboundary dissemination that poses a systemic threat to worldwide swine production [5].

While vaccination is theoretically the most cost-effective strategy for controlling the transmission of ASF, its practical implementation is hindered by the lack of a widely available, safe, and effective commercial vaccine. The ongoing global panzootic has intensified the demand for immunization, thereby accelerating research efforts [6]. Nevertheless, developing a viable vaccine remains an uphill battle due to the complexity of the large ASFV genome and the numerous proteins that facilitate host immune evasion [7,8]. Given these constraints, systematic epidemiological research becomes indispensable for the effective prevention and control of ASF. In particular, understanding the geographic distribution and movement of the virus is critical for identifying high-risk zones and optimizing resource allocation, which can be effectively achieved through spatial epidemiology. Spatial epidemiology harnesses geographic information systems (GIS) and spatial analysis techniques to extract and analyze information across spatial dimensions, thereby elucidating the distribution and evolutionary patterns of diseases and facilitating the development of more precise and sustainable control measures [9]. As spatial data becomes increasingly accessible, spatial epidemiology has emerged as a cornerstone in infectious disease research. Recently, the application of spatial statistical methods has gained considerable prominence in delineating disease distributions, identifying spatial clustering, and uncovering the complex relationships between environmental determinants and disease risk factors [10,11]. With the growing adoption and widespread integration of GIS and spatial analytical techniques in epidemiological studies, the potential to advance the understanding of animal disease systems and their underlying mechanisms has been significantly enhanced. However, spatial patterns alone provide only a static snapshot of the epidemic. To fully capture the evolving nature of ASF, it is essential to complement spatial insights with temporal dynamics, which is where time series analysis becomes pivotal.

Time series analysis entails extracting meaningful statistical patterns from chronologically ordered data [12]. While classical statistical models remain foundational, machine learning (ML), which enables algorithms to automatically learn predictive patterns from data, and deep learning (DL), which employs multilayer artificial neural networks to capture complex hierarchical features, have increasingly emerged as flexible and highly accurate approaches for the high-precision forecasting of infectious animal diseases [13,14,15]. In particular, ML algorithms such as RF, extreme gradient boosting (XGBoost), and support vector regression (SVR) are widely employed to model complex nonlinear dynamics in time series data [16,17]. Concurrently, DL architectures like gated recurrent units (GRU) and long short-term memory (LSTM) networks excel at capturing long-term temporal dependencies [18], while transformer-based models offer enhanced predictive capacity [17,19]. In recent years, time series modeling approaches have been widely applied in the prediction of infectious animal diseases, where they facilitate the characterization of epidemic dynamics and support the development of data-driven early warning systems.

Despite extensive research on the spatiotemporal epidemiology of ASF within specific regions [20,21,22,23,24,25], a comprehensive global-scale understanding remains limited by geographically confined scopes and a lack of integrated frameworks that combine spatial interaction analysis with predictive modeling. To fill this gap, this study integrates multi-distance spatial statistics with time series forecasting to characterize the global dynamics of ASF, elucidate the spatial interactions between domestic and sylvatic transmission cycles, and identify the most robust modeling framework for early warning systems.

To the best of our knowledge, this is the first study to integrate multi-distance spatial interaction analysis with a comprehensive comparative benchmark of eight time series forecasting models on a global scale. By synthesizing these two dimensions, we provide an unprecedented quantification of the spatiotemporal dynamics of ASF, uncovering transboundary transmission patterns that remain undetected in previous region-specific studies.

## 2. Materials and Methods

### 2.1. Data Collection

The ASF outbreak data was sourced from the Global Animal Disease Information System of the Food and Agriculture Organization of the United Nations (EMPRES-i+) (https://empres-i.apps.fao.org/). The data collection period spans from 1 April 1996 to 31 May 2025. The information collected included the time (observation date and report date) and location (region and country) of the ASF epidemic, species of infected animals (swine and wild boar), and the latitude and longitude coordinates. After cleaning and organizing the data, there were a total of 57,253 records remaining.

### 2.2. Exploratory Descriptive Analysis

An exploratory descriptive analysis was conducted to characterize the temporal and spatial distribution of ASF outbreaks. Data preprocessing was executed in R (version 4.5.1) through a structured pipeline: temporal variables were standardized to ISO 8601 format (YYYY-MM-DD) [26]; redundant records sharing identical geocoordinates and observation timestamps were excluded. Host classifications were manually validated, with misclassified wild boar outbreaks reassigned to the sylvatic category. Spatial patterns were quantified across hierarchical administrative levels, including region, subregion, and country. Furthermore, outbreaks were stratified by host type (domestic or wild animal) and further granularized at the species level. All statistical summaries and visualizations were generated using the ggplot2 package to elucidate the multifaceted distribution of ASF outbreaks.

### 2.3. Temporal Distribution of Global ASF Outbreaks

We evaluated temporal distribution by aggregating outbreak frequencies annually and monthly. This allowed us to characterize seasonality, delineate long-term evolution, and identify significant inflection points using Joinpoint regression (Joinpoint software 5.4.0.0; National Cancer Institute, Rockville, MD, USA). A log-linear model was constructed with the time unit defined as the independent variable and the outbreak count as the dependent variable. Data were log-transformed to facilitate the estimation of the Annual Percent Change (APC) for each detected trend segment and the Average Annual Percent Change (AAPC) for the entire study period. The optimal number of joinpoints was determined via a permutation test with a significance level of α = 0.05, which allowed the model to partition the epidemic trajectory into distinct temporal segments based on statistically significant inflection points. The statistical significance of each identified trend was rigorously evaluated using 95% confidence intervals (CIs). Specifically, a trend was categorized as “increasing” or “decreasing” when the APC significantly deviated from zero, which was characterized by a 95% CI strictly greater or less than zero, respectively.

### 2.4. Spatial Distribution of Global ASF Outbreaks

The geographic coordinates of ASF outbreak locations were recorded in the World Geodetic System 1984 (WGS 84) coordinate reference system, which is the standard coordinate system used in the FAO EMPRES-i+ database. Multi-distance spatial cluster analysis was conducted using Ripley’s K-function to identify the spatial distribution patterns of ASF. The analysis utilized a Python-based workflow (Python 3.13), calculating point distances via the Haversine formula to account for Earth’s curvature. To assess deviations from complete spatial randomness (CSR), the observed K-function, *K*(*r*), was transformed into variance-stabilized L-function, *L*(*r*) = $\sqrt{K(r)/\pi }$, and expressed as *L*(*r*)–*r*. Statistical significance was evaluated using Monte Carlo simulations under the assumption of CSR with 999 simulations to generate 95% confidence envelopes. Observed values exceeding the upper envelope were classified as significantly clustered. Results were visualized using matplotlib (version 3.9), plotting both *K*(*r*) and *L*(*r*)–*r* against distance r to pinpoint the specific scales of aggregation.

To systematically characterize the spatial heterogeneity and spreading dynamics of ASF, three integrated spatial statistical techniques were employed using ArcGIS 10.6 (ESRI, Redlands, CA, USA). First, kernel density estimation (KDE) was utilized to visualize spatial intensity and pinpoint high-risk clusters by generating a continuous surface of outbreak concentration within a specified search radius. Second, hotspot analysis (Getis-Ord Gi∗) was performed to detect statistically significant spatial clusters of infection. This method employed Z-scores and *p*-values to differentiate robust hot spots from random spatial patterns by evaluating outbreak frequencies relative to neighboring features. Finally, the directional distribution SDE tool was applied to quantify the spatial orientation, central tendency, and geographical dispersion of the epidemic. By calculating the mean center and standard deviations in x and y coordinates, SDEs were generated to delineate the principal axis and spatial extent of disease transmission over the study period.

### 2.5. Time Series Analysis

Monthly ASF outbreak data spanning from 2007 to 2025 was utilized for time series forecasting. To ensure data quality and suitability for modeling, comprehensive preprocessing steps were implemented: First, the Augmented Dickey-Fuller (ADF) test was conducted to assess the stationarity of the time series, which initially showed non-stationary characteristics (*p*-value > 0.05). To address this, first-order differencing was applied, transforming the series to achieve stationarity (*p*-value < 0.01). To preserve the temporal dependency structure of the data, the dataset was divided into training and testing sets using a chronological split rather than random sampling. Specifically, the earliest 80% of observations were used for model training, while the most recent 20% of observations were reserved for model evaluation. For DL models, the MinMaxScaler v0.17 was employed to normalize input features to the range [0, 1], enhancing model convergence and performance stability.

We utilized eight distinct modeling techniques, ranging from statistical methods including seasonal ARIMA (SARIMA) and Prophet, through ML algorithms (RF, XGBoost and SVR), to DL architectures comprising LSTM, GRU, and a hybrid convolutional neural network-LSTM (CNN-LSTM). For statistical models, SARIMA was optimized via grid search to identify the optimal non-seasonal (p, d, q) and seasonal (P, D, Q, s) parameters (s = 12 for monthly seasonality) based on the lowest Akaike Information Criterion (AIC). Prophet was configured with multiplicative seasonality to capture both trend and seasonal patterns. For ML models, hyperparameter optimization was performed using grid search combined with cross-validation to ensure methodological consistency and avoid overfitting. RF regressor (100 estimators) and XGBoost were trained using a sliding window approach to generate input features. For the SVR model, a radial basis function (RBF) kernel was adopted. The regularization parameter C and kernel coefficient gamma were optimized using grid search to identify the best-performing configuration. For DL models, LSTM and GRU networks (two layers with 50 hidden units each) were constructed to capture long-term dependencies, alongside a hybrid CNN-LSTM model that combined one-dimensional convolutional neural network (1D-CNN) for local feature extraction and LSTM for sequence modeling. Model performance was assessed using multiple predictive accuracy metrics, including the root mean square error (RMSE), mean absolute error (MAE), and mean absolute percentage error (MAPE). RMSE and MAE quantify the magnitude of prediction errors, while MAPE provides a scale-independent measure of relative forecasting accuracy. The model achieving the lowest RMSE and MAE values, together with consistently favorable MAPE scores, was considered to exhibit the best predictive capability.

## 3. Results

### 3.1. Global Distribution Characteristics of ASF

**Figure 1:**
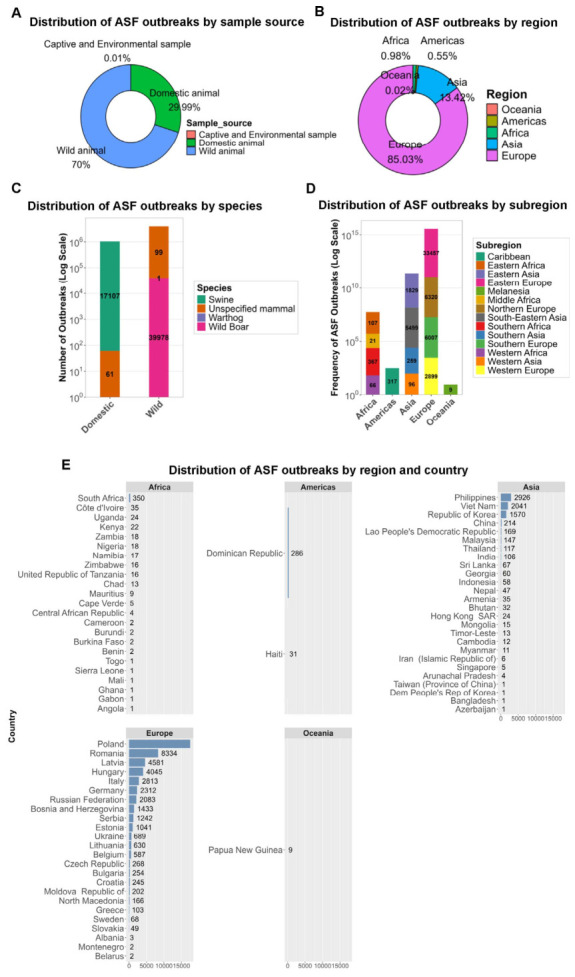
Global distribution characteristics of ASF. (**A**) Sample source analysis shows the dominance of the sylvatic cycle (70.00%) over the domestic compartment (29.99%), whereas captive and environmental samples represented only a negligible proportion (0.01%). (**B**) Europe (85.03%) and Asia (13.42%) represent the primary regional epicenters, while Africa (0.98%), the Americas (0.55%), and Oceania (0.02%) contributed comparatively few outbreaks. (**C**) Species frequency (log scale) confirms wild boar (Sus scrofa) and domestic swine as the distinct primary reservoirs; domestic swine accounted for 17,107 outbreaks; only sporadic cases were associated with warthogs (*n* = 1) and unspecified mammals (domestic: *n* = 61; wild: *n* = 99). (**D**) Subregional analysis (log scale) identifies Eastern Europe and South-Eastern Asia as the major epidemic hotspots, with additional contributions from Southern Europe, Northern Europe, Eastern Asia, and other affected subregions. (**E**) National outbreaks stratified by region highlight hyper-endemic nations, including Poland, Romania, and the Philippines; comparatively lower outbreak frequencies were observed in Africa, the Americas, and Oceania.

The global epidemiological landscape of ASF is characterized by a marked disparity between wild and domestic compartments (Figure 1A). Wild animals constituted the vast majority of recorded outbreaks, accounting for 70.00% of the total outbreaks, whereas domestic animals represented 29.99%; captive and environmental samples contributed a negligible fraction (0.01%). Granular analysis of host species further elucidates this divergence, particularly within the sylvatic cycle (Figure 1C). The wildlife compartment was overwhelmingly dominated by wild boar (Sus scrofa), involved in 39,978 outbreaks, while reports in warthogs (*n* = 1) and other unspecified mammals (*n* = 99) remained marginal. Conversely, the domestic sector recorded 17,168 outbreaks, centered almost exclusively on domestic swine (*n* = 17,107; >99.6%). These data indicate that while domestic outbreaks pose acute socio-economic threats, the global persistence and transboundary spread of ASF are fundamentally driven by infection dynamics within wild suid populations. Geographically, Europe is the most heavily impacted region, accounting for 85.03% of global outbreaks (Figure 1B). Subregional analysis reveals that this dominance is driven by Eastern Europe, which reported an overwhelming 33,457 outbreaks, followed by Northern and Southern Europe (Figure 1D). Asia represents the second most affected region at 13.42%, characterized by heavy viral pressure in Southeast Asia (*n* = 5499) and Eastern Asia (*n* = 1829). In contrast, Africa reported only 0.98% of outbreaks, primarily localized in Southern (*n* = 367) and Eastern Africa (*n* = 107). The Americas (0.55%) and Oceania (0.02%) maintained minimal notification levels, with outbreaks concentrated in the Caribbean and Melanesia, respectively. These data underscore a profound geographical imbalance, where the current panzootic of ASF is fundamentally centered in the Northern Hemisphere, particularly across Eastern Europe and Southeast Asia.

The national distribution of ASF outbreaks highlights a high concentration of viral activity within a limited number of territories (Figure 1E). Poland emerged as the most severely affected nation, reporting 17,531 outbreaks, which accounts for over 30% (30.62%) of the global total, followed by Romania (*n* = 8334) and Latvia (*n* = 4581). In Asia, the epidemic was primarily driven by the Philippines, which recorded 2926 outbreaks, significantly exceeding its regional counterparts such as Vietnam (*n* = 2041) and South Korea (*n* = 1570). Conversely, in the African and American regions, the disease burden was largely localized in South Africa (*n* = 350) and the Dominican Republic (*n* = 286), respectively. The dominance of ASF outbreaks in Eastern European countries, particularly within wild boar populations, underscores the role of specific national landscapes in sustaining the current panzootic.

### 3.2. Temporal Dynamics of ASF Outbreaks

**Figure 2:**
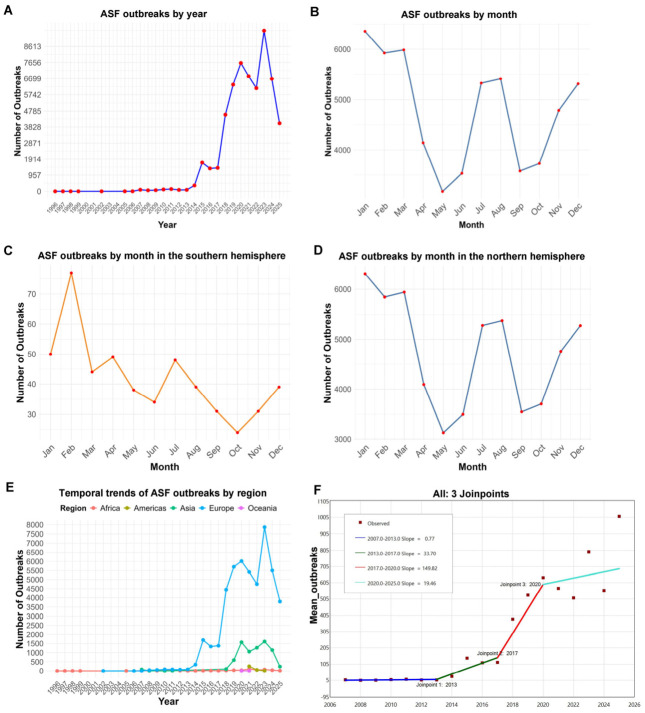
Temporal trends and seasonality of ASF outbreaks (1996–2025). (**A**) Annual outbreaks show a dramatic escalation post–2014. (**B**–**D**) Seasonal decomposition indicates (**B**) a global bimodal pattern (winter/summer peaks), predominantly driven by (**D**) the Northern Hemisphere, in contrast to (**C**) the distinct, low-intensity cycle of the Southern Hemisphere. (**E**) Regional time series demonstrate the dominance of the European epidemic wave (blue) and the subsequent Asian emergence (green). (**F**) Joinpoint regression analysis reveals three statistical inflection points (2013, 2017, 2020), defining four distinct phases of viral acceleration (slopes). Red squares and lines indicate observed means and modeled trends, respectively.

The temporal evolution of ASF outbreaks from 1996 to 2025 exhibited a marked upward trajectory, characterized by distinct phases of acceleration. As illustrated in Figure 2A, the global incidence remained relatively low and stable prior to 2014, followed by a precipitous surge that peaked around 2023–2024. This observation was statistically corroborated by Joinpoint regression analysis (Figure 2F), which identified three significant inflection points in the time series, partitioning the study period into four progressive epidemiological phases with varying growth rates. The trend was characterized by a period of latency (2007–2013, Slope = 0.77), followed by a moderate increase (2013–2017, Slope = 33.70), and a phase of rapid acceleration (2017–2020, Slope = 149.82). Although the incidence continued to rise post-2020, the rate of increase appeared to attenuate (Slope = 19.46). These findings underscore a significant transition from sporadic localized outbreaks to a sustained global panzootic, with the 2017–2020 window representing the most critical period of viral acceleration.

Regarding seasonal distribution, global ASF outbreaks displayed a clear bimodal pattern (Figure 2B), with a primary peak occurring in the first quarter (January–March) and a secondary elevation during the summer months (July–August). A distinct trough was observed in May. Notably, this global seasonality was predominantly driven by the Northern Hemisphere (Figure 2D), which showed a high degree of concordance with the overall global pattern in both magnitude and trend. In contrast, the Southern Hemisphere (Figure 2C) exhibited a significantly lower outbreak magnitude (max < 80 outbreaks/month) and a divergent seasonal rhythm, peaking in February with varying fluctuations throughout the year.

Geographically, the burden of ASF outbreaks demonstrated substantial regional heterogeneity over the study period (Figure 2E). Europe emerged as the dominant epicenter, driving the global surge observed after 2014, with annual outbreaks reaching roughly 8000 at the peak. Asia exhibited a secondary but significant wave of infection initiating around 2018, coinciding with the rapid acceleration phase identified in the Joinpoint analysis. Conversely, the reported outbreak numbers in Africa, the Americas, and Oceania remained comparatively low and stable relative to the surges observed in Eurasia.

### 3.3. Spatial Distribution characteristics of Global ASF Outbreaks

**Figure 3:**
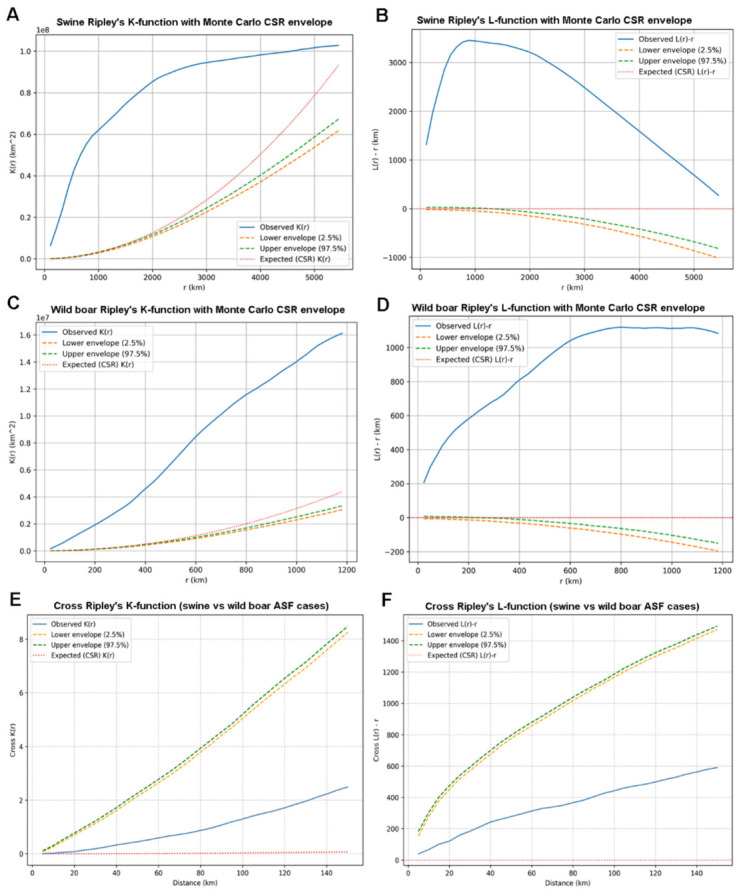
Multi-distance spatial cluster analysis of global ASF outbreaks using Ripley’s K- and L-functions. Observed *K*(*r*) and normalized *L*(*r*) curves (blue lines) are compared against complete spatial randomness (CSR) (red dotted line) and 95% Monte Carlo simulation envelopes (dashed lines). (**A**–**D**) Univariate plots for (**A**,**B**) swine and (**C**,**D**) wild boar outbreaks consistently show significant clustering above the upper simulation envelopes. (**E**,**F**) In contrast, bivariate Cross–function analysis indicates spatial segregation between domestic swine and wild boar compartments at local scales (0–150 km), with curves falling below the lower envelopes.

Ripley’s K- and L-function analyses revealed significant spatial clustering of ASF outbreaks in both swine and wild boar populations. For swine, the observed *K*(*r*) and *L*(*r*)–*r* curves consistently exceeded the upper Monte Carlo CSR envelopes across nearly all evaluated distances, indicating strong spatial aggregation rather than complete spatial randomness. The clustering intensity increased rapidly at short to intermediate distances and remained above the confidence envelope at broader spatial scales, suggesting large-scale spatial concentration of outbreaks within the domestic swine compartment (Figure 3A,B). A similar pattern was detected for wild boar outbreaks, where the observed functions were substantially higher than the simulated envelopes, confirming that wild boar outbreaks also exhibit significant clustering, although the aggregation appeared more localized and concentrated at shorter spatial distances (Figure 3C,D).

To further evaluate the spatial interaction between outbreaks in swine and wild boars, Ripley’s cross-K and cross-L functions were applied. The results show that the observed cross-K curve lies consistently below the lower Monte Carlo confidence envelope, indicating significant spatial segregation between outbreaks occurring in swine and wild boars at short-range distances (0–150 km) (Figure 3E). This pattern is also confirmed by the cross-L function results, where the observed *L(r)–r* values remain below the lower confidence envelope across the analyzed distance range (Figure 3F).

**Figure 4:**
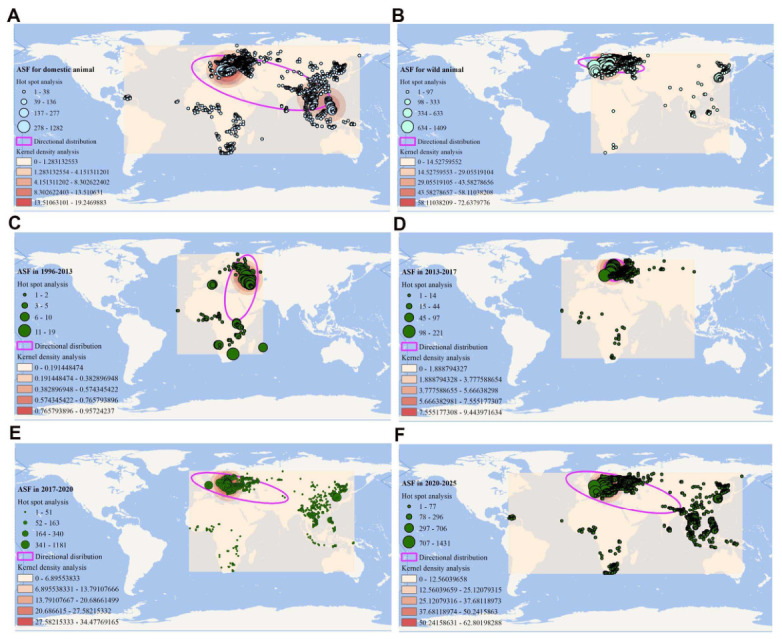
Spatiotemporal evolution of ASF outbreaks using kernel density and directional distribution. Comparison of host compartments shows (**A**) widespread trans-Eurasian clustering in domestic animals versus (**B**) European localization in wild animals. Temporal trajectory analysis reveals a progressive shift from (**C**) an early meridional spread pattern during 1996–2013, to (**D**) intensified clustering and northward concentration in Eastern Europe during 2013–2017, followed by (**E**,**F**) a broad zonal trans-Eurasian expansion from 2017–2025. Pink SDEs indicate the directional trend and dispersion; background gradients and green circles represent viral density and local hot spots, respectively.

KDE and hot spot analysis revealed pronounced spatial heterogeneity between epidemiological cycles. For domestic animals, high-density clustering was observed in two distinct transcontinental epicenters: Eastern Europe and Southeast Asia, resulting in a highly elongated SDE spanning the Eurasian landmass (Figure 4A). Conversely, the sylvatic cycle exhibited a unimodal high-density cluster strictly confined to Central and Eastern Europe, with the SDE indicating a more localized spatial footprint (Figure 4B).

The temporal evolution of the epidemic trajectory demonstrated a significant directional shift over the study period. Between 1996 and 2013, the directional distribution was oriented along a meridional (North–South) axis, reflecting transmission between Africa and the Caucasus region (Figure 4C). The 2013–2017 period was characterized by a consolidation of high-density hotspots within Eastern Europe (Figure 4D). A critical spatial inflection occurred during 2017–2020, where the SDE underwent a dramatic zonal expansion along the west–east axis, driven by the rapid transboundary dissemination of the virus into East and Southeast Asia (Figure 4E). By 2020–2025, the spatial pattern stabilized into a vast, continuous trans-Eurasian belt, with sustained high-density hotspots persisting across both European and Asian territories (Figure 4F).

### 3.4. Results of Time Series Analysis

The comparative analysis of ASF epidemic time series models utilized monthly outbreaks data spanning January 2007 to April 2025 (*n* = 220 observations), demonstrating substantial temporal variability with outbreaks ranging from 0 to 1367 (mean ± SD: 260.18 ± 308.33). Stationarity assessment via ADF testing indicated non-stationary characteristics (*p*-value > 0.05), necessitating first-order differencing to achieve stationarity. The dataset was stratified into training (80%, *n* = 176) and testing (20%, *n* = 44) subsets for model validation.

**Figure 5:**
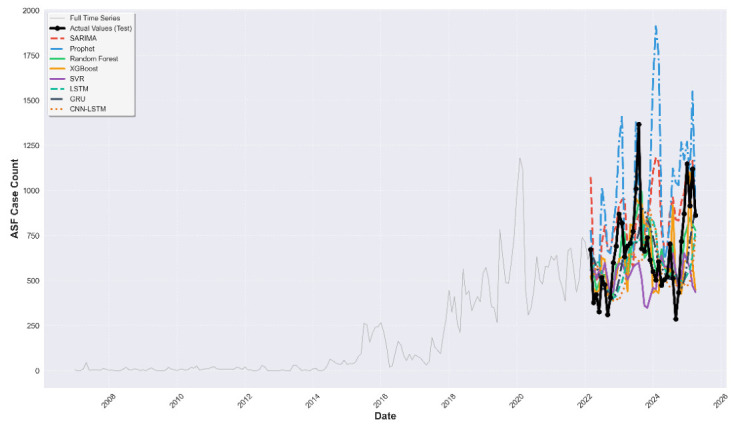
Comparative performance analysis of predictive models for global ASF outbreaks. The thin gray line represents the historical time series (training set, 2007–2022) used for model development. The thick black line with markers denotes the ground truth (observed actual values) during the hold-out test period (2022–2025). Colored trajectories illustrate the forecasting results generated by three distinct modeling categories: traditional statistical methods (SARIMA, Prophet), ML algorithms (RF, XGBoost, SVR), and DL architectures (LSTM, GRU, CNN-LSTM). Visual inspection reveals the superior fit of ensemble-based ML models (e.g., RF, green line) in capturing the volatility of the test data compared with statistical baselines like Prophet (blue dashed line), which exhibited significant overestimation.

Time series forecasting results show notable differences in model performance in capturing the temporal dynamics of ASF outbreaks (Figure 5). The full time series revealed a marked increase in outbreak frequencies after approximately 2017, followed by pronounced fluctuations and multiple peaks during the testing period. Although most models successfully reproduced the overall upward trend and seasonal patterns, their predictive accuracy differed markedly. Evaluation on the hold-out test set using RMSE, MAE, and MAPE established a clear performance hierarchy. ML approaches demonstrated superior accuracy, effectively capturing both long-term trends and intermediate fluctuations, with RF achieving the best performance (RMSE = 174.3254, MAE = 142.5808, MAPE = 22.8799%), closely followed by XGBoost (RMSE = 221.5554, MAE = 175.4708, MAPE = 26.2540%). The SVR model produced comparatively conservative predictions with reduced sensitivity to abrupt changes (RMSE = 270.1758, MAE = 211.6260, MAPE = 31.0669%). DL architectures exhibited competitive but variable efficacy. Among them, the GRU model (RMSE = 223.2218, MAE = 171.3740, MAPE = 26.2635%) performed nearly on par with XGBoost, whereas LSTM and the hybrid CNN-LSTM showed moderate results (MAPE ~29–30%). Traditional statistical models significantly lagged behind modern algorithmic approaches. While SARIMA captured the overall trend, it smoothed short-term fluctuations, resulting in reduced accuracy (RMSE = 293.4757, MAPE = 45.2992%). The Prophet displayed greater variability and, in some instances, overestimated peak magnitudes, which produced the largest prediction error across all metrics (RMSE = 469.4987, MAPE = 63.5605%) (Table 1). Overall, the results underline the dominance of ML methods, particularly RF with sliding window feature construction, over DL and statistical baselines for modeling ASF incidence trends.

## 4. Discussion

ASF has evolved from a regional concern into a devastating global panzootic, representing one of the most significant threats to the contemporary swine industry and global food security [4]. Characterized by high environmental resistance and mortality rates approaching 100% in swine, the disease imposes profound socio-economic repercussions, including massive herd depopulation, trade embargoes, and the destabilization of pork supply chains, particularly in major producing regions across Eurasia [26]. Despite the urgent demand for pharmaceutical interventions, the commercial availability of safe and effective vaccines remains limited, leaving control strategies heavily reliant on stringent biosecurity protocols, early detection, and “stamping out” policies [27]. The complexity of ASF eradication is further exacerbated by the virus’s capacity to persist in wild suids and its transmission through anthropogenic routes, underscoring the critical need for continuous epidemiological surveillance to monitor its transboundary expansion [28,29]. Therefore, understanding the spatiotemporal dynamics and host-specific patterns of ASF is essential for refining these control strategies.

Wildlife reservoirs play a critical role in the spread and persistence of ASF [5,30]. Our analysis confirms that the current ASF panzootic is fundamentally driven by a self-sustaining sylvatic cycle, particularly in the Northern Hemisphere where wild animals account for the vast majority of outbreaks (70%). This ecological shift renders conventional stamping-out policies insufficient, as wildlife reservoirs maintain viral circulation independently of domestic populations. In addition, the pronounced dominance of wild boar in sustaining ASF transmission, particularly in Eastern Europe, underscores the critical role of the sylvatic cycle as a persistent viral reservoir. This phenomenon can be attributed to the high density of wild boar populations and their extensive foraging ranges, which facilitate the rapid, undetected spread of the virus across natural landscapes. Unlike domestic swine, which are subject to strict biosecurity and movement controls, wild boar populations create an open, uncontrolled transmission network. The observed spatial interaction between these two systems suggests that the wildlife–livestock interface acts as a ‘leaky barrier,’ where periodic spillover events from wild reservoirs re-trigger outbreaks in domestic herds. This dynamic explains the recurrent nature of ASF in several regions, where eradication in domestic populations is consistently undermined by the environmental persistence of the virus within wild boar populations. Consequently, effective control now hinges on managing the wildlife–livestock interface to prevent recurrent spillover.

The temporal dynamics revealed in our study characterize ASF as an evolving panzootic with distinct acceleration phases. The rapid acceleration period identified by Joinpoint analysis temporally coincides with the virus’s transcontinental expansion into Asia [31]. It likely reflects the interaction of multiple epidemiological processes across different regions. On the one hand, sustained transmission within wild boar populations in Europe has been widely recognized as a major driver of long-term ASF persistence and outbreak accumulation. On the other hand, the introduction of ASF into high-density domestic swine populations in East and Southeast Asia during this period resulted in large-scale outbreaks that significantly amplified the global burden of the disease. Together, these parallel transmission dynamics across wildlife and domestic compartments likely contributed to the rapid rise in reported outbreaks observed during this period. Notably, the subsequent attenuation in growth rate post-2020 should not be misinterpreted as disease disappearance; rather, it likely indicates a transition from an epidemic invasion phase to an endemic status in affected Eurasian regions, where host depletion and adaptive biosecurity have stabilized transmission.

Furthermore, the pronounced bimodal seasonality driven by the Northern Hemisphere highlights the duality of ASF transmission pathways. The winter peak (Jan–Mar) could potentially be associated with increased anthropogenic activities, including swine movements, trade, and management practices that may facilitate virus transmission among domestic herds, while the summer peak may be partly related to ecological dynamics within wild boar populations, such as seasonal changes in behavior, population structure, or potential vector activity [32,33]. Additionally, the discordance between Northern and Southern Hemisphere patterns confirms that global ASF epidemiology is currently dictated by Eurasian ecosystem dynamics. Consequently, risk mitigation strategies must be seasonally adaptive, intensifying biosecurity during these high-risk windows.

Our spatial analyses reveal a critical dichotomy in ASF epidemiology, where the reduced cross-type spatial interaction between domestic swine and wild outbreaks implies that these compartments operate as distinct epidemiological subsystems driven by divergent transmission mechanisms. In many regions, the persistent circulation of ASF in wild boar populations is likely driven by ecological factors including habitat suitability, population density, and carcass-mediated transmission, which collectively foster the localized clustering of outbreaks [34]. In contrast, outbreaks in domestic swine often occur within livestock production networks shaped by farm distribution, biosecurity practices, and human-mediated activities. Previous epidemiological studies have suggested that long-distance anthropogenic transport, including the movement of swine, pork products, and contaminated equipment, may contribute to the dissemination of ASF across large geographic distances [35,36]. However, because the present study did not explicitly incorporate transportation network data or related infrastructure variables, this interpretation should be considered a potential explanatory mechanism supported by existing literature rather than a direct inference from our analysis. Future studies integrating epidemiological surveillance data with transportation and trade network information may help further clarify the relative roles of wildlife ecology and anthropogenic mobility in shaping ASF spatial dynamics.

Regarding time series analysis, RF is well recognized for its ability to reduce overfitting and variance through ensemble mechanism [17]. In this study, the superior performance of RF highlights the necessity of robust ensemble approaches for modeling the non-linear temporal dependencies of ASF, which proved inadequate for traditional linear time series models. The relatively weaker performance of the Prophet model may reflect limitations of its decomposable trend–seasonality framework in capturing structural changes in ASF outbreak data, which may be better represented by more flexible ML models. Meanwhile, the comparable efficacy of XGBoost and GRU confirms the utility of both gradient boosting and recurrent neural networks for sequential data. Interestingly, the moderate results of SVR and the hybrid CNN-LSTM model indicate that neither kernel-based methods nor complex hybrid architectures offered additional benefits over pure ensemble or sequential models. Consequently, prioritising tree-based ML methods appears most effective for enhancing the predictive accuracy of ASF early warning systems. Nevertheless, the current analysis focused solely on temporal patterns, excluding critical exogenous variables (e.g., livestock movements, biosecurity measures, and climate factors). Future research could integrate these variables to enhance predictive accuracy and explore hybrid models that combine statistical methods’ seasonal awareness, ML’s nonlinear capture capabilities, and DL’s proficiency in sequential modeling.

## 5. Conclusions

In summary, our integrated spatiotemporal framework offers a first-of-its-kind comprehensive understanding of the global ASF panzootic, highlighting the divergence between domestic and sylvatic transmission dynamics. The identification of the RF algorithm as the most robust predictive tool underscores the importance of incorporating non-linear machine learning approaches into infectious disease surveillance. By elucidating the critical role of wild boar reservoirs and the shifting transboundary dissemination patterns, this work establishes a quantitative foundation for optimizing early warning systems and refining global control strategies.

## Figures and Tables

**Table 1:** Evaluation results of time series models.

	RF	XGBoost	SVR	GRU	LSTM	CNN-LSTM	SARIMA	Prophet
RMSE	174.3254	221.5554	270.1758	223.2218	251.1807	278.3153	293.4757	469.4987
MAE	142.5808	175.4708	211.6260	171.3740	191.8617	209.4201	238.9996	346.0406
MAPE	22.8799	26.2540	31.0669	26.2635	28.9574	29.9799	45.2992	63.5605

## Data Availability

The data presented in this study were derived from the following resources available in the public domain: https://empres-i.apps.fao.org/. The raw datasets and analytical code are publicly available at https://github.com/lirenfeng2018/ASF_analysis.git (accessed on 1 May 2026).
